# A study on the factors influencing university students’ subjective well-being based on machine learning methods

**DOI:** 10.1186/s40359-026-04415-7

**Published:** 2026-03-28

**Authors:** Yunkun Zhang, Muhammad Syawal Amran, Hazrati Husnin, Lanlan Yin

**Affiliations:** 1 Hechi University, Yizhou, Guangxi 546300 China; 2 Faculty of Education, Universiti Kebangsaan Malaysia, Bandar Baru Bangi , Selangor 43600 Malaysia

**Keywords:** Subjective well-being, Family functioning, Psychological risk factors, Machine learning methods

## Abstract

Subjective well-being is a key area of research in psychology. Based on survey data from 384 university students, this study employed automated machine learning methods to construct a predictive model of subjective well-being, in which the Support Vector Machine (SVM) model performed best, achieving an overall prediction accuracy of 81.81%. The results indicate that the overall subjective well-being of the current university student population is relatively high; depression and interpersonal sensitivity are the most significant influencing factors, followed by hostility and paranoid ideation, among others. Family emotion also has a significant impact on subjective well-being. Based on these findings, fostering positive psychological traits and optimizing family functioning are suggested as approaches to enhance university students’ subjective well-being, thereby promoting their academic achievement and mental health development.

## Introduction

In recent years, mental health issues such as depression and anxiety among university students have increasingly garnered societal attention. Subjective well-being (SWB), as a key indicator for assessing mental health, reflects an individual’s affective and cognitive evaluation of their own quality of life [1]. To comprehensively understand the influencing mechanisms of subjective well-being, this study constructs a multi-level and interactive theoretical framework. This framework integrates Self-Determination Theory(SDT), Family Systems Theory(FST), and Conservation of Resources Theory(COR). Self-Determination Theory, starting from individuals’ intrinsic psychological needs, posits that the fulfillment of autonomy, competence, and relatedness constitutes the foundation of well-being [2]. Family Systems Theory, from an interpersonal perspective, reveals how family interaction patterns, emotional climate, and other systemic functions shape the psychological states of its members [3]. As a core model within this theoretical framework, the McMaster Model of Family Functioning further posits that family interaction patterns and emotion are key determinants of members’ mental health and well-being [4].Conservation of Resources Theory offers a perspective for understanding dynamic processes, conceptualizing well-being as a psychological resource whose level is influenced by spiral of resource gain and loss [5]. The family functioning, psychological risk factors, and demographic variables examined in this study correspond respectively to the individual background, external systems, and internal states that affect resource accumulation and depletion. This multi-level framework provides a unified theoretical foundation for subsequent variable selection and the interpretation of complex relationships. Notably, high levels of well-being can lead to stronger motivation, significantly predicting academic engagement and achievement [6, 7], thereby further promoting individual development and social adaptation.

Subjective well-being, as a multidimensional and complex variable, is influenced by the interactive effects of various factors. Existing research indicates that family functioning is a key external factor affecting an individual’s subjective well-being [8]. Based on the McMaster Model of Family Functioning, the quality of family functioning directly affects the psychological well-being of its members [9]. For example, the efficiency of problem-solving, the appropriateness of emotional involvement, and the clarity of communication within the family all influence individuals’ emotional experiences and quality of life [10]. Difficulties at the family system level often lead to psychological distress in individuals, while a harmonious family atmosphere can significantly enhance members’ well-being [3]. Parents’ provision of moderate support is positively correlated with positive emotional experiences and can also effectively reduce the frequency of negative emotions [11]. This support needs to maintain a balanced level, as excessive support may decrease well-being [12]. Furthermore, different dimensions of family functioning have differentiated effects on well-being. Research by Xiang et al. [8] confirmed that good family functioning and positive coping strategies for difficulties can positively predict subjective well-being. In contrast, when family member relationships are estranged or family functioning is disrupted (manifesting as chaotic interaction patterns), individuals’ well-being levels significantly decrease [13]. These studies reveal the core role of the family system in shaping individual well-being, and thus, the complex relationship between family functioning and well-being needs to be understood from a systematic and dynamic perspective.

A large body of research indicates that psychological risk factors such as depression, interpersonal relationships, hostility, and paranoia significantly reduce university students’ subjective well-being [14, 15]. This finding highlights the importance of enhancing well-being in the prevention of mental health disorders, especially for high-risk groups, such as individuals with schizophrenia, who typically experience lower well-being and life satisfaction [16]. Studies show that individuals with depression often exhibit frequent negative emotional experiences and lower life satisfaction [17]. A 10-year longitudinal study found that the level of well-being in individuals without depression/anxiety disorders was approximately twice that of those with such conditions [18]. Sensitivity in interpersonal relationships is negatively correlated with subjective well-being, and interpersonal communication skills are also negatively correlated with well-being [19]. Good interpersonal relationships can significantly enhance subjective well-being, while interpersonal difficulties can have a negative impact [20]. Hostility and paranoia are closely related, with the two reinforcing each other at the cognitive, emotional, and behavioral levels. Hostile emotions are not only significantly associated with poor health [21] but also further decrease subjective well-being by affecting interpersonal interactions [22]. In social situations, hostility can easily lead to communication barriers and conflicts [23], especially when individuals perceive their happiness, social status, or goals to be threatened, which further triggers hostile responses. It is noteworthy that subjective well-being can buffer paranoia tendencies. For example, Thompson et al. [24] found that interventions based on well-being helped reduce paranoid thinking and improve the ability to maintain interpersonal relationships in adolescent groups. This finding suggests that enhancing well-being may serve as an effective way to alleviate hostility and paranoia. Although existing research confirms that these psychological risk factors reduce subjective well-being, there is currently limited comparative analysis of their relative impact strength.

Additionally, research indicates that individual characteristics significantly influence subjective well-being. Gender and age differences are particularly notable: women tend to experience negative emotions more frequently than men [25, 26], while older students generally report lower levels of subjective well-being compared to their younger counterparts [26]. Family background factors—including household income [27], parental education level, marital status [28], and occupation [29, 30]—are also closely associated with well-being. Specifically, family income shows a positive correlation with happiness, whereas students from low-income families often face greater financial stress, resulting in diminished well-being [31–33]. Academic discipline further contributes to variations in subjective well-being. For instance, students in humanities and social sciences typically report higher well-being scores than those in science and engineering fields [34].

Although existing studies have extensively explored the influence of family functioning and psychological risk factors on subjective well-being, systematic comparisons of the relative importance of these factors remain limited, and few studies have employed machine learning methods to capture their nonlinear effects and interaction patterns. Therefore, this study applies machine learning algorithms to quantify the contribution of each influencing factor and uncover the complex relationship patterns between these factors and well-being, thereby addressing gaps in the current literature.

With the rapid advancement of artificial intelligence, machine learning has provided a novel methodological perspective for psychological research due to its superior predictive capabilities [35, 36]. Its application in well-being research has become increasingly widespread in recent years [7, 37], offering a new pathway for investigating complex, multi-determined psychological phenomena such as subjective well-being.This study employs machine learning not merely to pursue higher predictive accuracy, but specifically to address several limitations inherent in traditional statistical models when examining complex psychological constructs. Traditional psychology research has predominantly relied on explanatory modeling, emphasizing unbiased parameter estimation and causal inference. However, such approaches often encounter challenges in handling multivariate interactions, nonlinear relationships, and applied prediction tasks.To address these gaps, this study introduces a predictive modeling framework. Its key advantages are threefold: First, in terms of predictive efficacy and generalizability, machine learning algorithms are optimized for out-of-sample prediction accuracy. They can identify robust, predictive feature patterns from a multitude of variables, which holds direct practical value for the future development of model-based screening tools for well-being. Second, regarding the capture of complex relationships, well-being is influenced by factors that often interact in nonlinear or multiplicative ways. Machine learning algorithms, free from assumptions of linearity, can more flexibly model the intricate associations among variables, thereby offering a closer approximation to the true underlying psychological mechanisms. Third, concerning interpretability—a common critique of machine learning’s “black-box” nature—this study integrates explainable artificial intelligence techniques such as SHAP (Shapley Additive Explanation) and PDP (Partial Dependence Plot). SHAP values quantify the contribution of each feature to the prediction, while PDP visualize the dynamic relationship between a feature and the predicted outcome. Together, they not only assess variable importance but also elucidate the direction and form of their effects. This combined approach maintains predictive performance while revealing interaction patterns and boundary conditions that are difficult to visualize using traditional regression analysis, thereby addressing the psychological field’s concern for model transparency and explanatory depth.Furthermore, compared to traditional methods (e.g., multiple linear regression), machine learning offers distinct advantages: it is more adept at detecting the nonlinear relationships and interaction effects that are common yet often undetected by linear models in psychological data. When handling high-dimensional psychological datasets, machine learning can automatically mitigate issues of multicollinearity and identify key predictors along with their relative importance without relying on manual variable selection.

Based on this, the present study comprehensively employs six algorithms, including Logistic Regression, Random Forest, XGBoost, LightGBM, Deep Neural Networks, and Support Vector Machine (SVM), covering multiple paradigms such as traditional statistical models, ensemble learning, and deep learning. By comparing different modeling strategies, the study ensures the robustness and predictive performance of the results. It systematically investigates the impact of variables across three dimensions—demographics, family functioning, and psychological risk factors—on subjective well-being. The best-performing model was selected and further utilized, with SHAP values employed to assess the relative importance of each factor and PDP used to visualize the specific predictive relationships between key variables and well-being. This approach not only enhances predictive accuracy but also deepens the understanding of the mechanisms through which influencing factors operate.

## Methods

### Participants

A total of 387 university students from two universities in Guangxi and Hebei participated in the study, with 384 valid questionnaires collected. Among the participants, 253 were male (66%) and 131 were female (34%). The participants’ ages ranged from 17 to 22 years. Specifically, 126 were first-year students, 133 were second-year students, 122 were third-year students, and 3 were fourth-year students. Before the survey was conducted, all respondents were informed about the purpose and content of the study and provided their informed consent.

### Research instruments

#### Demographic questionnaire

This study employed a demographic questionnaire to collect general information from undergraduate students at universities in Hebei and Guangxi. The questionnaire encompassed the following variables: Gender、Age、Ethnicity、Family residence location、Grade、Family economic status、Average monthly living expenses at university、Frequency of family contact (monthly)、Parental education level、Parental marital status.

#### Family function scale

The Family Function Assessment Form, developed by Smilkstein et al. from the University of Washington in the United States, was adopted. It was mainly used to evaluate an individual’s family function, including five items such as fitness, cooperation, growth, emotion and intimacy of family function [38]. A three-level scoring method is employed, with a total score range of 3 to 15 points. Scores of 3 to 6 indicate severe dysfunction in family functioning, 7 to 10 denote moderate dysfunction, and 11 to 15 signify good family functioning.The scoring method is shown in Table 4. Studies show that this questionnaire has good reliability and validity. In this study, the reliability and validity values of this scale are 0.88 and 0.86 respectively, indicating that the internal consistency among various items is relatively good.

#### Symptom checklist-90 (SCL-90)

The Symptom Checklist-90 (SCL-90), originally developed by Derogatis [39], was employed to assess psychological symptom patterns. This 90-item self-report inventory measures ten primary symptom dimensions: Somatization、Obsessive-Compulsive、Interpersonal Sensitivity、Depression、Anxiety、Hostility、Phobic Anxiety、Paranoid Ideation、Psychoticism、Additional items (sleep and eating disturbances). A five-point rating scale was employed, with the specific scoring method detailed in Table 4. Scores across each dimension were categorised according to normative data into three groups: asymptomatic (≤ 2 points), mild (2–3 points), and moderate to severe (≥ 3 points). In the current study, the scale demonstrated exceptional psychometric properties with a Cronbach’s α coefficient of 0.98 for reliability and a validity index of 0.97, confirming excellent internal consistency among all items.

#### Subjective well-being scale

The subjective well-being scale developed by Chinese scholar Xing Zhanjun [40] was used, which includes 10 dimensions and 20 items: goal value experience, physical health experience, contentment and abundance experience, psychological health experience, growth and progress experience, attitude balance experience, social confidence experience, interpersonal relationship experience, self-acceptance experience, and family atmosphere experience. The scoring method is shown in Table 4. In this study, the reliability and validity of the scale were 0.88 and 0.89, respectively.

#### Model prediction methods

The core concept of the logistic regression model involves achieving multiclass classification of data through maximum likelihood estimation.Random Forest, an ensemble learning method, enhances prediction accuracy and stability by constructing multiple decision trees and combining their outputs. This approach effectively handles high-dimensional data while demonstrating strong resistance to overfitting.XGBoost, another ensemble technique based on boosting principles, employs iterative rounds of gradient descent approximation to progressively optimize the loss function and approach the optimal solution.LightGBM represents an efficiency-optimized implementation of the traditional Gradient Boosted Decision Trees algorithm, with core improvements focusing on computational speed and memory efficiency while maintaining high predictive accuracy.Deep Neural Networks utilize multiple layers of nonlinear transformations to automatically learn hierarchical feature representations from data, enabling modeling of complex relationships. SVM though technically a feedforward neural network architecture, demonstrate unique advantages in addressing small-sample, nonlinear, and high-dimensional pattern recognition problems. Empirical studies confirm their widespread applicability in psychological research.

## Data analysis

### Data processing

The specific data processing steps are as follows: First, data cleaning and encoding. Data cleaning primarily involves imputing missing values with the mean. Data encoding primarily involves performing 0, 1, 2 classification encoding on the label column to transform it into numerical data required by machine learning models.Next, model training. In order to achieve the best prediction accuracy, this study uses six machine learning algorithms for model training: Logistic Regression, Random Forest, XGBoost, LightGBM, Deep Neural Networks, and SVM. To ensure the validity of the models and improve prediction accuracy, 5-fold cross-validation and grid search are applied during the training process to optimize hyperparameters.Finally, model evaluation and application. The accuracy, recall, and other evaluation metrics for each model are compared to identify the optimal model. Using the trained model, the impact of different feature values on subjective well-being is analyzed (importance ranking of features), the relationship between individual features and well-being, and the relationship between multiple features and well-being. The model is used to measure the overall well-being of university students, providing data support for the education department to develop precise intervention strategies.

### Model performance evaluation of different methods

To obtain the optimal parameters for the models, the dataset was randomly divided into a training set and a test set at an 8:2 ratio. Through hyperparameter optimization, the best predictive performance of each model was evaluated and obtained. In classification tasks, the calculation of each metric is based on the classification results of positive and negative samples, represented by a confusion matrix, as shown in Table 1 below.

**Table 1:** Confusion matrix

Predicted Value Actual Value	Positive	Negative
Ture	TP(Ture Positive)	TN(Ture Negative)
False	FP (False Positive)	FN(False Negative)

Here, TP represents the number of true positives, TN denotes the number of true negatives, FP stands for the number of false positives, and FN indicates the number of false negatives.

Based on the confusion matrix, various performance metrics can be calculated to comprehensively evaluate the model. The evaluation methods for the accuracy of the trained model are shown in Table 2 below:

**Table 2:** Multiple evaluation metrics for models

Evaluation Method	Calculation Formula
The proportion of correctly predicted samples to the total samples	$\:\mathrm{A}\mathrm{c}\mathrm{c}\mathrm{u}\mathrm{r}\mathrm{a}\mathrm{c}\mathrm{y}=\frac{TP+TN}{TP+FP+TN+FN}$
The proportion of true positives among samples predicted as positive	$\:\mathrm{P}\mathrm{r}\mathrm{e}\mathrm{c}\mathrm{i}\mathrm{s}\mathrm{o}\mathrm{n}=\frac{TP}{TP+FP}$
The proportion of correctly predicted positives among all actual positive samples	$\:\mathrm{R}\mathrm{e}\mathrm{c}\mathrm{a}\mathrm{l}\mathrm{l}=\frac{TP}{TP+FN}$
The harmonic mean of Precision and Recall	$\:F1=\:\frac{2\left(\mathrm{P}\mathrm{r}\mathrm{e}\mathrm{c}\mathrm{i}\mathrm{s}\mathrm{o}\mathrm{n}\times\:\mathrm{R}\mathrm{e}\mathrm{c}\mathrm{a}\mathrm{l}\mathrm{l}\right)}{\left(\mathrm{P}\mathrm{r}\mathrm{e}\mathrm{c}\mathrm{i}\mathrm{s}\mathrm{o}\mathrm{n}\mathrm{+}\mathrm{R}\mathrm{e}\mathrm{c}\mathrm{a}\mathrm{l}\mathrm{l}\right)}$

The performance evaluation results of the six machine learning algorithms are shown in Table 3. In terms of accuracy, the performance of the six algorithms was as follows: SVM (81.81%) was the best, followed by logistic regression (80.51%), with Random Forest and XGBoost tied at (77.92%), Deep Neural Networks (66.23%), and LightGBM (62.34%). Across all four evaluation metrics, the SVM demonstrated superior performance. The Support Vector Machine slightly outperforms Logistic Regression in terms of accuracy, F1 score, and other metrics. It also demonstrates more stable performance in cross-validation, particularly achieving a more balanced F1 score for minority groups in subjective well-being classification. Therefore, this study employs SVM.

**Table 3:** Evaluation results of different machine learning algorithm models

	Logistic Regression	Random Forest	XGBoost	LightGBM	Deep Neural Networks	SVM
Accuracy	80.51	77.92	77.92	62.34	66.23	81.81
Recall	80.51	77.92	77.92	79.37	66.23	81.81
Precision	80.59	78.16	79.37	77.92	76.55	82.84
F1	80.27	77.38	77.72	77.72	67.35	81.50

### Evaluation results of feature importance

**Fig. 1:**
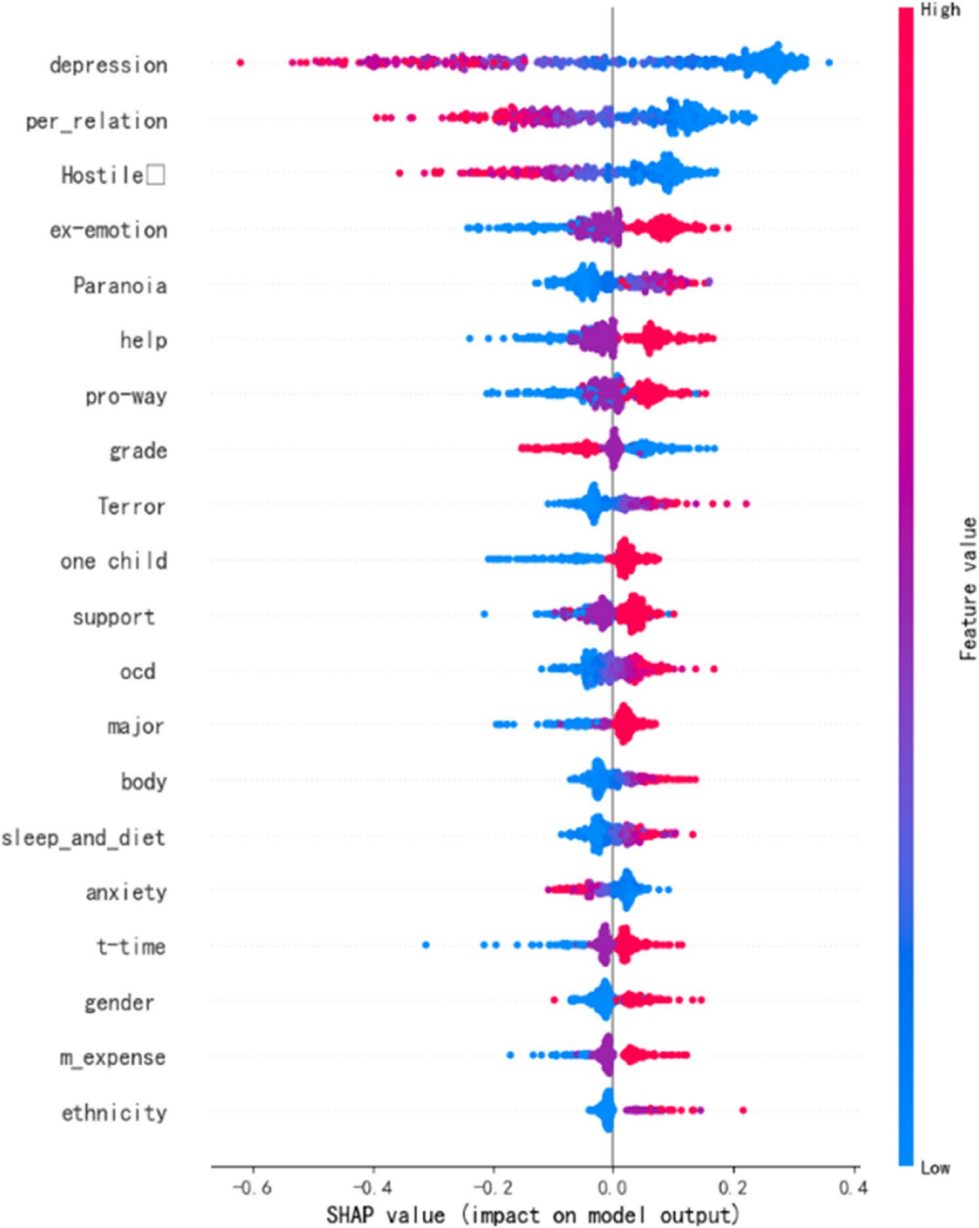
Feature importance

Based on the machine learning iteration results, 27 features were ultimately retained for model training. The encoding methods and results are presented in Table 4. The target variable represents subjective well-being, with the following classifications: 0 for low well-being (0–40), 1 for moderate well-being (41–80), and 2 for high well-being (81–120) [41]. The features include depression, income level, age, major, etc. Table 4 displays the ranking of key features by their importance, where higher importance indicates stronger explanatory power for the dependent variable. As shown in Table 4; Fig. 1, depression is the most influential feature affecting university students’ subjective well-being, followed by interpersonal sensitivity, hostility, family emotion, paranoia, and others. The following sections will focus on analyzing the impact of several important individual features on university students’ subjective well-being.

**Table 4:** Feature importance ranking

Feature No.	Feature Name	Description & Encoding Method	Feature Importance Coefficient
15	depression	Depression: None = 1, Mild = 2, Moderate = 3, Relatively Severe = 4, Severe = 5.	0.2200
20	per_relation	interpersonal sensitivity: None = 1, Mild = 2, Moderate = 3, Relatively Severe = 4, Severe = 5.	0.1202
14	Hostile	Hostility: None = 1, Mild = 2, Moderate = 3, Relatively Severe = 4, Severe = 5.	0.0925
21	ex-emotion	Satisfaction with Family Emotion Expression: Rarely = 1, Sometimes = 2, Often = 3.	0.0655
16	Paranoia	Paranoia: None = 1, Mild = 2, Moderate = 3, Relatively Severe = 4, Severe = 5.	0.0550
17	help	Satisfaction with the help received from family: Rarely = 1, Sometimes = 2, Often = 3.	0.0520
13	pro-way	Satisfaction with the way family discusses issues: Rarely = 1, Sometimes = 2, Often = 3.	0.0458
26	Grade	Grade: Freshman = 1, Sophomore = 2, Junior = 3, Senior = 4.	0.0438
22	Terror	Phobic Anxiety: None = 1, Mild = 2, Moderate = 3, Relatively Severe = 4, Severe = 5.	0.0390
19	one child	Only child: Yes = 1, No = 2.	0.0384
12	support	Received support from family in engaging in new activities: Rarely = 1, Sometimes = 2, Often = 3.	0.0353
25	ocd	Obsessive-compulsive: None = 1, Mild = 2, Moderate = 3, Relatively Severe = 4, Severe = 5.	0.0340
18	major	Major: Humanities = 1, Science = 2, Engineering = 3, Medicine = 4.	0.0332
23	body	Somatization: None = 1, Mild = 2, Moderate = 3, Relatively Severe = 4, Severe = 5.	0.0288
9	sleep_and_diet	Sleep and diet: None = 1, Mild = 2, Moderate = 3, Relatively Severe = 4, Severe = 5.	0.0286
1	anxiety	Anxiety: None = 1, Mild = 2, Moderate = 3, Relatively Severe = 4, Severe = 5.	0.0281
7	t-time	Satisfaction with time spent with family: Rarely = 1, Sometimes = 2, Often = 3.	0.0278
24	gender	Gender: Male = 1, Female = 2.	0.0274
4	m_expense	Average monthly living expenses at school: <500 RMB/month = 1, 500 ~ 1000 RMB/month = 2, > 1000 RMB/month = 3.	0.0266
8	ethnicity	Ethnicity: Han = 1, Zhuang = 2, Other = 3.	0.0205
10	age	University student age	0.0161
5	p_marital	Parental marital status: Unstable = 1, Stable = 2.	0.0140
0	address	Family residence: Rural = 1, Urban = 2.	0.0112
6	p_edu	Parental education level: Junior high school or below = 1, High school = 2, University = 3, Graduate school or above = 4.	0.0106
11	y_income	Family annual income: ≤ 20,000 RMB = 1, 20,000 ~ 50,000 RMB = 2, 50,000 ~ 100,000 RMB = 3, ≥ 100,000 RMB = 4.	0.0103
3	psychotic	Psychotic level: None = 1, Mild = 2, Moderate = 3, Relatively Severe = 4, Severe = 5.	0.0099
2	frequecy	Frequency of contact with family per month: <1 time/month = 1, 2 ~ 4 times/month = 2, 4 ~ 8 times/month = 3, > 8 times/month = 4.	0.0080

The feature importance in the above table can be visualized using SHAP plots. Red-colored features increase the predicted value (positive correlation), while blue-colored features decrease the predicted value (negative correlation). Purple indicates proximity to the mean value. Moreover, the wider the color band, the greater the influence of that particular feature.

## Results

### Key findings

This study systematically investigated the subjective well-being of university students and its relationship with family functioning, psychological risk factors, and demographic variables by employing a combination of questionnaire surveys and machine learning methods. The main findings are as follows: First, the distribution of subjective well-being levels among current university students in China is as follows: 52.08% reported high well-being, 46.36% reported moderate well-being, and 1.56% reported low well-being. Second, all predictor variables significantly predicted subjective well-being, with psychological risk factors and family functioning identified as important influencing factors, which is consistent with existing research conclusions [17, 42 ].

### Predictive effects of key feature variables

The results of the machine learning modeling show that the Support Vector Machine (SVM) is the optimal predictive model. On the test set, it achieved an accuracy of 81.81%, with recall and F1 scores of 81.81% and 81.50, respectively, outperforming the other five comparative models. Further analysis of variable importance based on Shapley values indicates that all predictor variables contribute significantly to the model. Among these, the top five variables in order of importance are: depression, interpersonal sensitivity, hostility, family emotion and paranoia.

**Fig. 2:**
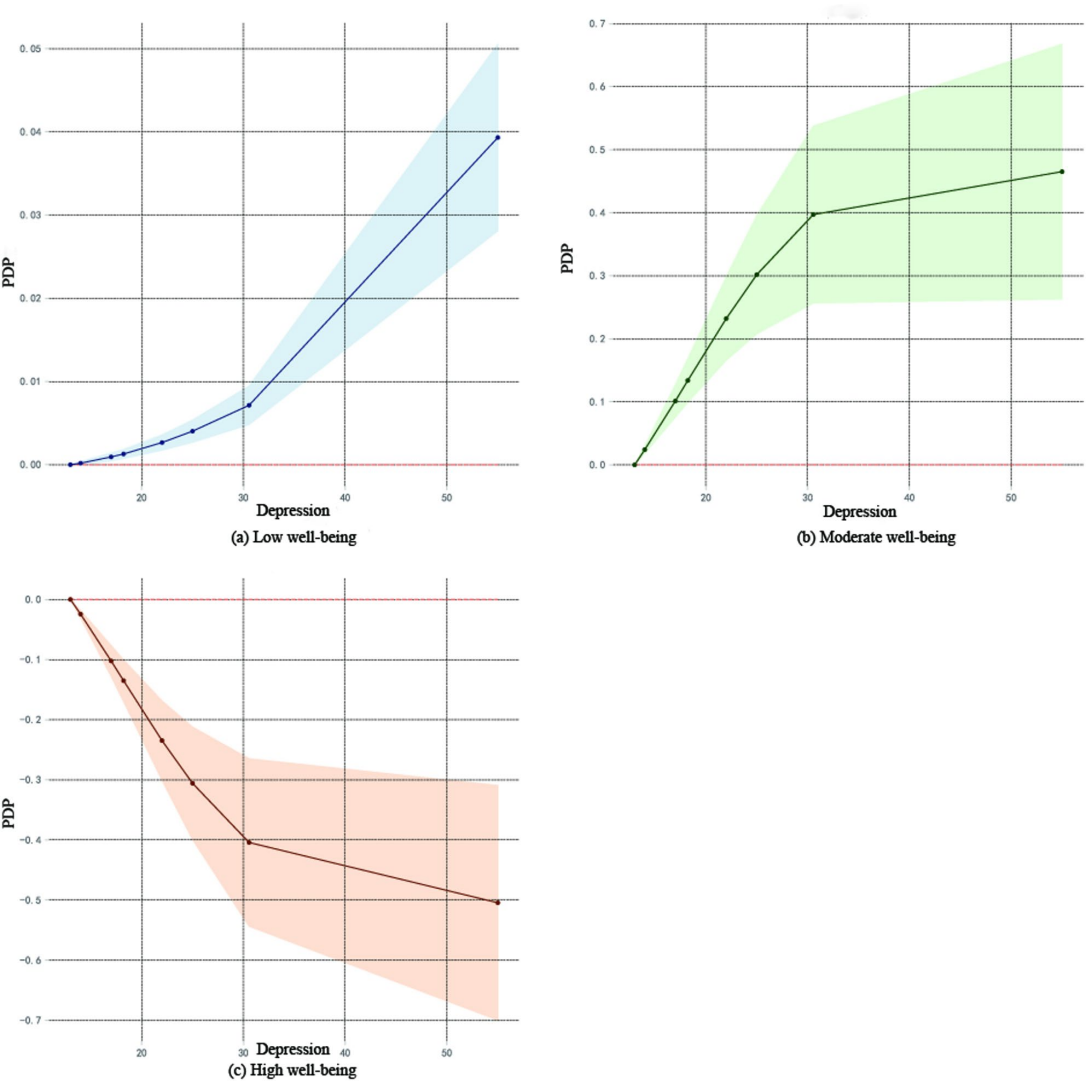
Depression and subjective well-being

**Fig. 3:**
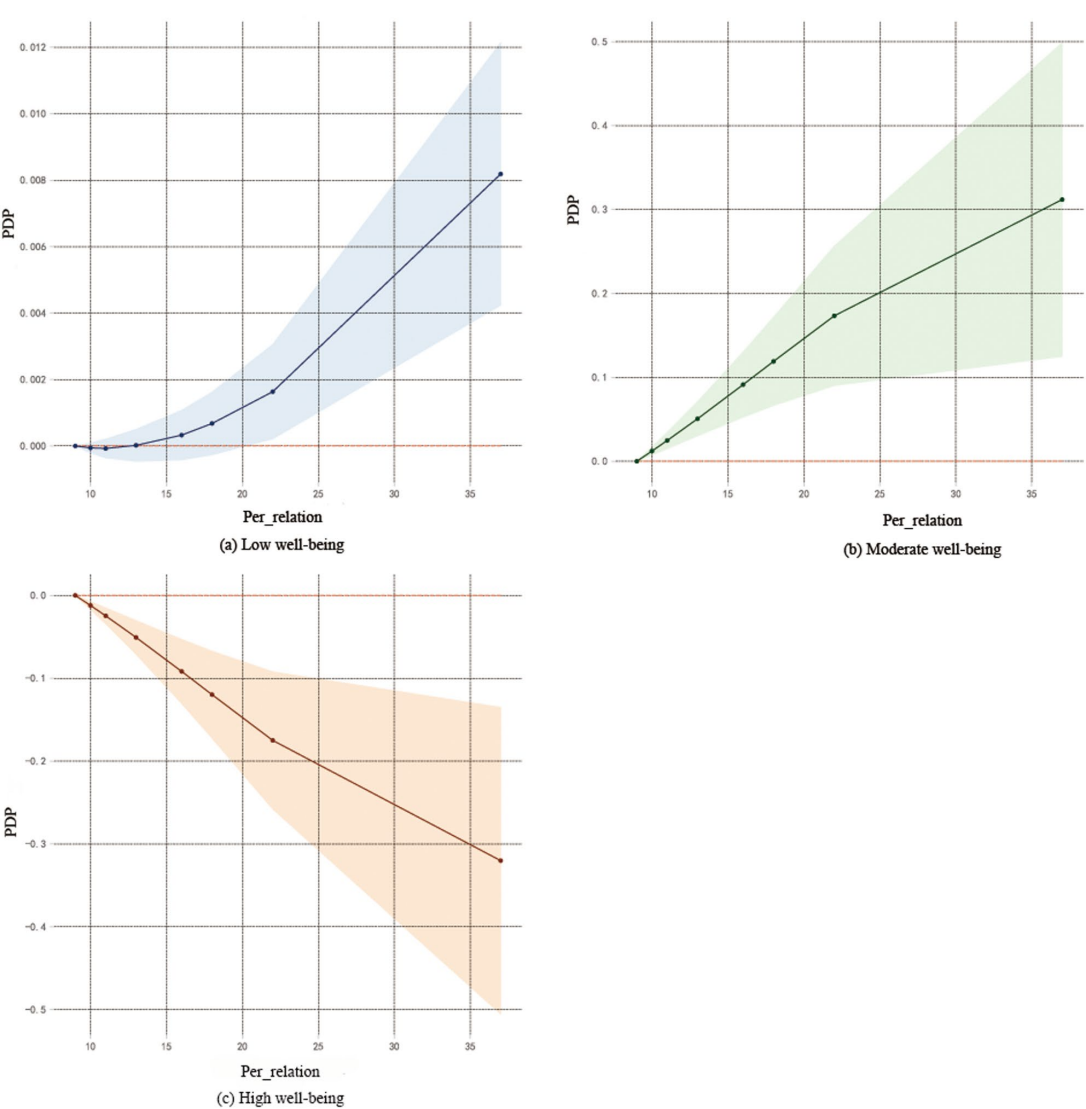
Interpersonal sensitivity and subjective well-being

**Fig. 4:**
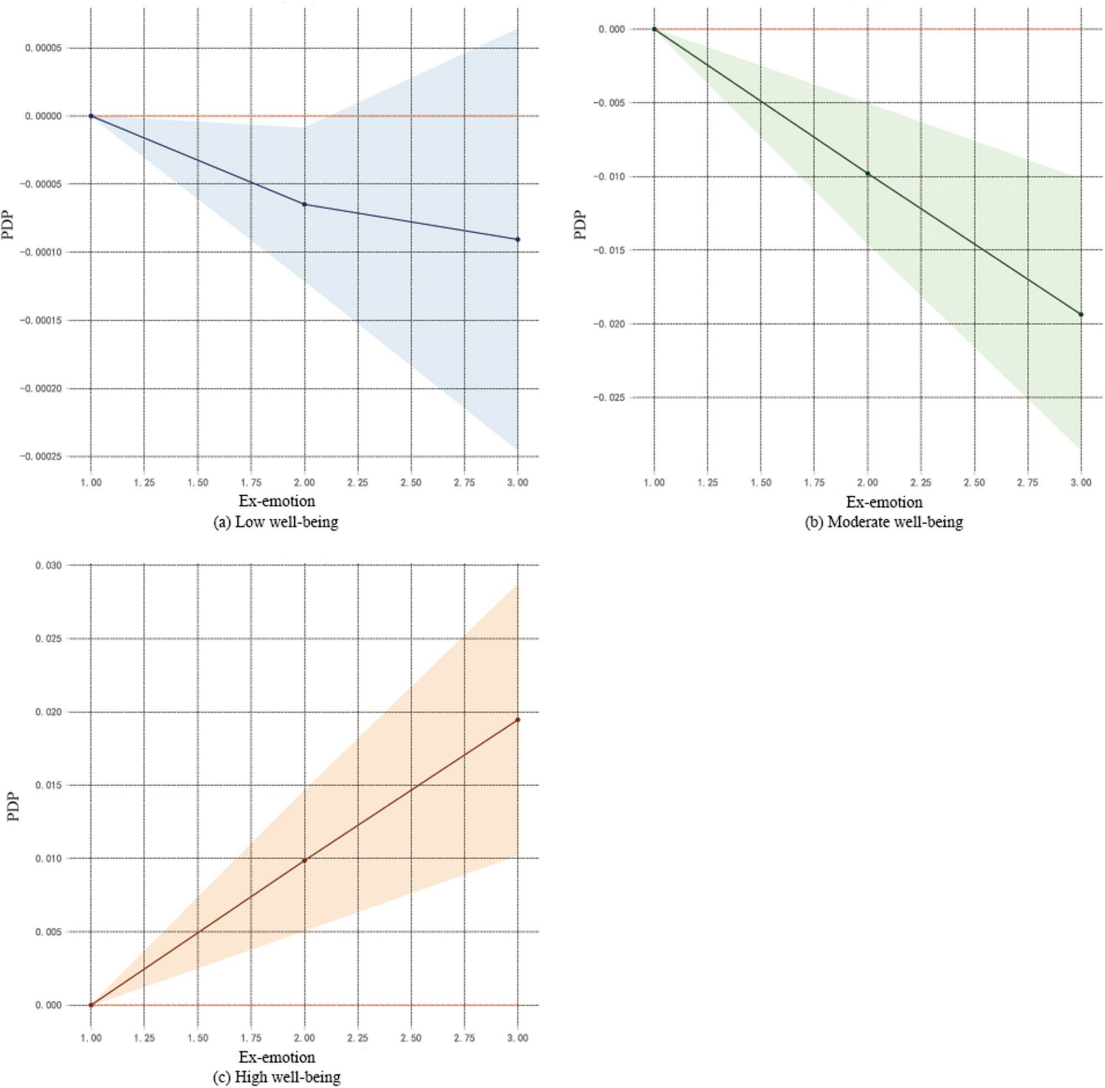
Family emotion and subjective well-being

**Fig. 5:**
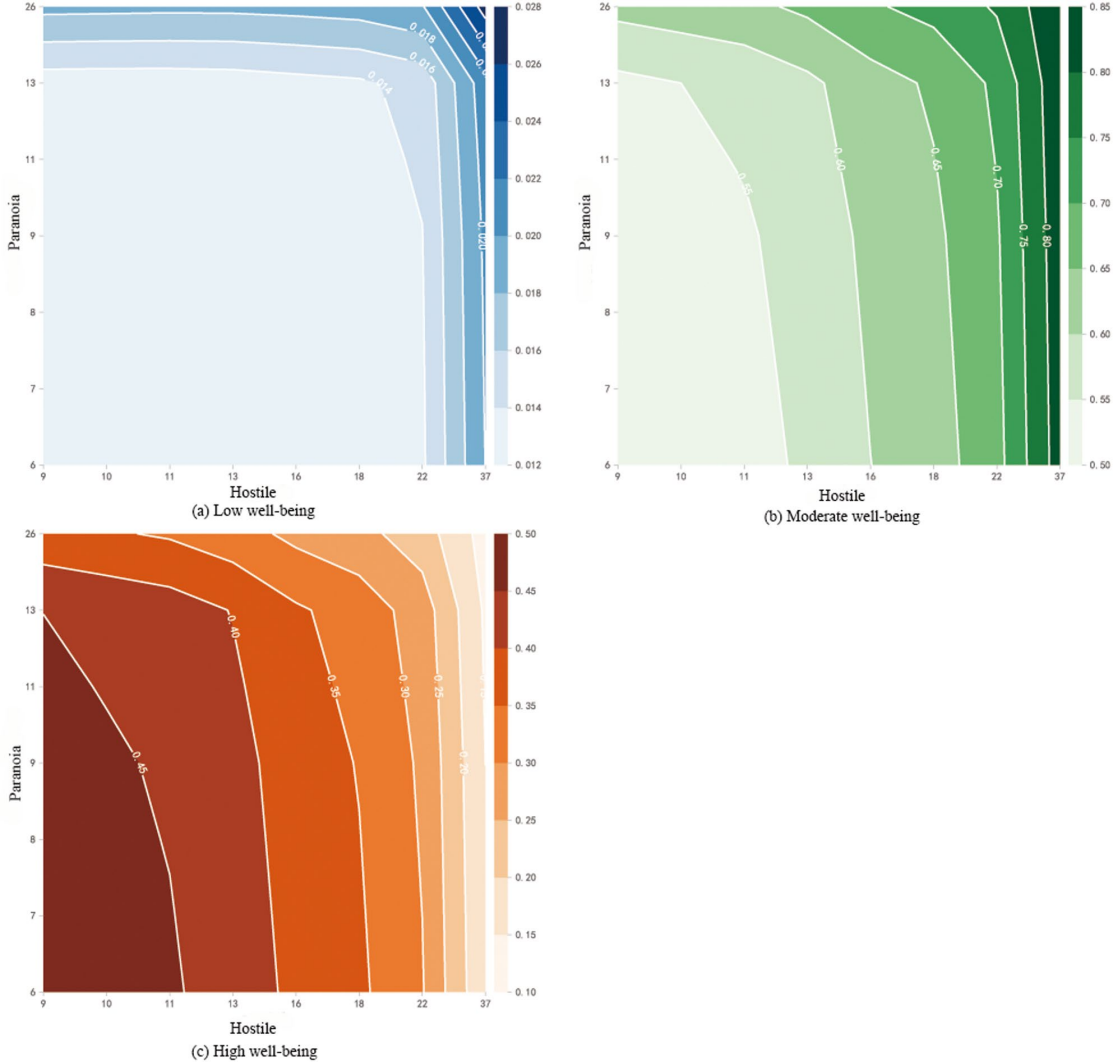
The impact of bivariate interaction on subjective well-being

To further reveal the complex relationships between key variables and well-being, this study conducted a visual analysis using PDP.Depression (Fig. 2), Among the high well-being group, depression showed a negative correlation with well-being, while in the moderate and low well-being groups, it exhibited a positive correlation.Interpersonal sensitivity (Fig. 3), Similar to the pattern for depression, interpersonal sensitivity was negatively correlated with well-being in the high well-being group but positively correlated in the moderate and low well-being groups.Family emotion (Fig. 4): In the high well-being group, family emotion was positively correlated with well-being, whereas in the moderate and low well-being groups, it showed a negative correlation.Hostility and Paranoia (Fig. 5), bivariate interaction analysis revealed that both hostility and paranoia were positively correlated with well-being in the moderate and low well-being groups but negatively correlated in the high well-being group, with highly consistent trends of change.The above results indicate that core variables such as depression, interpersonal sensitivity, family emotion, hostility and paranoia exhibit nonlinear and context-dependent association patterns with the subjective well-being of university students. The direction of their effects varies depending on the individual’s initial level of well-being. 

## Discussion

The optimal model (SVM) identified in this study achieved a predictive accuracy of 81.81%. In the field of mental health research, this level of accuracy holds substantial practical significance, indicating that the model can classify the subjective well-being levels of over 80% of university students with relatively high reliability based on limited questionnaire information. This provides a feasible basis for developing rapid and low-cost preliminary screening tools, which could facilitate early identification of individuals with relatively low well-being and potential need for psychological support within university populations. More importantly, the SHAP analysis revealed core influencing factors—such as depression, interpersonal sensitivity, and family emotion—thereby highlighting priority targets for the design of precise and tiered intervention strategies. For instance, intervention resources could be strategically allocated to these high-impact factors to enhance overall intervention efficiency. It is noteworthy that the relationships between these core factors and well-being are not simply linear but exhibit complex, context-dependent patterns.

### Theoretical explanation of influencing factors

#### Depression and subjective well-being

Depression has been confirmed as a key predictor of subjective well-being, consistent with previous studies [17]. The visualization of PDP reveals a non-linear relationship between depression and subjective well-being. Specifically, among individuals with high well-being, depression exhibits a negative correlation with subjective well-being. This may be attributed to their relatively abundant psychological and social resources, with depressive symptoms rapidly depleting key psychological resources such as positive cognitive styles. According to the “loss spiral” effect of Conservation of Resources theory, individuals with greater initial resources often experience more pronounced negative impacts when resources are diminished. Consequently, high well-being individuals commonly exhibit a marked decline in subjective well-being when experiencing depression.In contrast, among individuals with moderate to low well-being, depression shows a positive correlation with subjective well-being. One possible explanation is that these individuals, already in a state of relative resource scarcity, may have their resource conservation mechanisms activated by depressive emotions, prompting them to actively seek external support from family or friends. This process can facilitate the adjustment and restructuring of internal resources, ultimately leading to a moderate recovery in well-being. However, from a family systems theory perspective, this phenomenon could also reflect a pathological symbiotic mechanism. Within dysfunctional family systems, an individual’s depressive emotions may serve as a tool for maintaining a maladaptive equilibrium—for example, by assuming a “sick role” to elicit excessive attention and care from family members, thereby generating a transient and distorted sense of “satisfaction.” Such dependency-based “adaptation” does not constitute genuine psychological growth and may instead result in long-term impairment of individual autonomy. Therefore, whether this represents a true “protective mechanism” warrants careful evaluation, taking into account family interaction patterns and long-term developmental outcomes.

#### Interpersonal sensitivity and subjective well-being

Interpersonal sensitivity is also an important factor influencing subjective well-being, consistent with previous studies [19]. Analysis of the PDP reveals a context-dependent nonlinear relationship between interpersonal sensitivity and subjective well-being: at low to moderate levels of well-being, interpersonal sensitivity exhibits a positive correlation with well-being; at high levels of well-being, however, this relationship becomes negative, further illustrating the complexity of its underlying mechanism. Individuals with high well-being typically possess broad and stable social networks. When interpersonal sensitivity increases, they may overinterpret others’ intentions, leading to sustained cognitive resource depletion. Concurrently, socially avoidant behaviors resulting from heightened sensitivity can also diminish effective utilization of social support, thereby eroding well-being. Conversely, among individuals with moderate to low well-being, an appropriate degree of interpersonal sensitivity may prompt closer observation of the social environment, enabling the identification and proactive acquisition of potential support resources, which may contribute to a short-term improvement in well-being. This finding aligns with the Conservation of Resources theory, which posits that individuals dynamically adjust and compensate under conditions of resource scarcity.However, caution is warranted in conceptually interpreting the positive association between interpersonal sensitivity and well-being at lower well-being levels as an “adaptive” or “protective” mechanism. From the perspective of “enmeshed pathology” in family systems theory, an excessive sensitivity to external evaluations may reflect blurred interpersonal boundaries or emotional over-involvement, which may not be adaptive in the long term. Rather, it may sustain or even exacerbate psychological burdens within a dysfunctional family system. Therefore, future studies should incorporate longitudinal designs and qualitative methods to further clarify the mechanisms through which interpersonal sensitivity operates at different levels of well-being and its long-term psychosocial adaptation outcomes.

#### Family emotion and subjective well-being

According to the importance ranking results derived from the full sample above, family emotion is also an important factor influencing subjective well-being, consistent with previous studies [8]. Analysis reveals that the relationship between family emotion and well-being exhibits a non-linear pattern depending on an individual’s level of happiness: at high levels of well-being, the two are positively correlated; whereas at medium to low levels of well-being, they are negatively correlated. Specifically, individuals with high happiness levels typically recognise and integrate family emotion effectively, transforming it into psychological resources to create a virtuous cycle where emotional nourishment and happiness mutually reinforce each other. Conversely, those with moderate to low happiness levels, constrained by their own resource limitations, may perceive high emotional investment from family as pressure or a burden, thereby further diminishing their sense of well-being. This finding suggests that when implementing family interventions, it is crucial to consider the individual’s baseline level of well-being and carefully assess the intensity of support provided, to avoid counterproductive outcomes arising from “over-support”.

#### The impact of bivariate interaction on subjective well-being

Hostility and paranoia, as important predictive variables, are significantly correlated with subjective well-being. This finding is consistent with existing research [43, 44]. Despite the strong correlation between hostility and paranoia (*r* = 0.88), this study employed a bivariate interaction PDP model to examine their combined effect pattern, thereby mitigating the interpretative limitations posed by multicollinearity. While SHAP was used to assess their individual contributions, PDP visualizations illustrated their synergistic effects after controlling for other variables. The results indicated that in the low-to-moderate well-being group, both hostility and paranoia showed a positive correlation with subjective well-being, whereas in the high well-being group, the correlation was negative, with consistent trends in both cases. This confirms that hostility and paranoia jointly influence well-being in a synergistic manner.The underlying mechanisms may be explained as follows: For individuals with high well-being, who typically exhibit positive cognitive biases, the negative cognitions associated with hostility and paranoia may trigger internal conflict, thereby reducing subjective well-being. Conversely, in the low-to-moderate well-being group, an increase in hostility and paranoia was associated with elevated well-being, which may be attributed to psychological defense mechanisms. Through external attribution and emotional catharsis, individuals may alleviate pressures arising from self-criticism, temporarily enhance their sense of control, and consequently experience a short-term improvement in subjective well-being.However, beyond being interpreted as a defense mechanism, the observed “positive correlation” in the low-to-moderate well-being group may also reflect a learned maladaptive relational pattern. In family environments characterized by prolonged conflict or dysfunctional communication, hostility and paranoia may become default coping strategies. In certain contexts, they may even provide a transient sense of control or a distorted sense of belonging, thereby manifesting statistically as a positive correlation with well-being. From the perspective of family systems theory, this phenomenon is more likely to represent a form of “pathological cohesion” rather than a truly adaptive mechanism.Therefore, future longitudinal research may clarify whether elevated hostility and paranoia in low well-being contexts constitute a protective psychological strategy or, instead, reflect deeper maladaptation and impaired relational functioning.

### Demographic variables and their impact on subjective well-being

Factors such as academic year, whether one is an only child, and major also have a certain predictive effect on subjective well-being. While the predictive effects of these variables are relatively weak, their importance in the model’s prediction remains significantly above 0. For example, academic year significantly influences subjective well-being, consistent with existing research [45]. Freshman students, who are just entering university, may face significant adaptation pressures, such as adjusting to a new environment, academic requirements, which can impact their well-being. In contrast, senior students typically have already adapted to university life, with their academic and life rhythms becoming more stable, leading to higher subjective well-being.

### Practical implications

University students are at a crucial developmental stage in early adulthood. Families, schools, and society should fully respect their individual personality traits and promote their healthy growth in the following ways: First, cultivate self-awareness and psychological resilience. University students should be encouraged to deepen their self-awareness, explore their personal potential, learn to transform setbacks into growth opportunities, and establish a growth mindset. The accumulation of these positive psychological resources can effectively resist negative cognition and harmful behavior patterns, thereby preventing the occurrence of mental health issues.Second, strengthen the family support system. At the family level, university students can be guided to participate in collaborative family affairs and jointly set family goals to enhance family cohesion. This positive interaction provides essential psychological support for university students, fostering their healthy development.

### Research limitations

This study has several limitations that should be acknowledged. First, the predictive model of university students’ subjective well-being achieved an explained variance of 81.81%, leaving approximately 19% of the variance unexplained. Future research could optimize the model through the following approaches: theoretically, by exploring additional potential predictors to enrich the model structure; methodologically, by leveraging the capacity of machine learning for large-scale data—for instance, transforming the current questionnaire into an app- or web-based format to expand sample size, incorporate external validation sets, and perform parameter tuning to further enhance predictive accuracy.Second, regarding data collection, the current study relied primarily on self-reported measures of subjective well-being, which may be susceptible to subjective bias. Future studies could integrate multimodal data sources—such as behavioral experiments, ecological momentary assessment, and physiological indicators—to enable complementary validation and improve the objectivity and validity of the findings.Third, as the study is based on cross-sectional data, it cannot capture the dynamic processes underlying well-being. Longitudinal designs or time-series analyses are recommended in future research to examine the long-term trajectories and developmental patterns of subjective well-being.Finally, the sample was drawn from only two provinces. Although it reflects certain regional characteristics, caution is warranted when generalizing the findings to the broader national population of university students. While the current sample size (*N* = 384) and cross-validation procedures contributed to model stability, the limited sample may constrain the model’s generalizability. Subsequent research would benefit from multi-center collaborations to increase sample representativeness and from validation using independent external datasets to enhance the reliability and generalizability of the model.

## Conclusion

This study focuses on university students’ subjective well-being and systematically examines the key factors influencing it and their relative importance using machine learning algorithms. The results show that the predictive model obtained through machine learning algorithms has an accuracy of 81.81%, enabling a relatively accurate prediction of university students’ subjective well-being. Overall, university students’ subjective well-being largely depends on their own psychological risk factors and family functionality. These findings provide a scientific basis for the theoretical construction of subjective well-being development in university students and early intervention for psychological risks. Additionally, this study also demonstrates that machine learning, as a core method of artificial intelligence, is an effective approach for analyzing developmental data.

## Data Availability

The datasets used and/or analyzed during the current study are available from the corresponding author on reasonable request.

## Abbreviations

XGBoost: EXtreme Gradient Boosting
LightGBM: Light Gradient Boosting Machine
SCL: Symptom Check List
SVM: Support Vector Machine
